# Implications and prospects of immunological and neuroimaging research in HIV-associated neurocognitive disorders

**DOI:** 10.3389/fneur.2026.1699610

**Published:** 2026-02-20

**Authors:** Xing-Yuan Jiang, Chuan-Ke Hou, Ju-Ming Ma, Jiao-Jiao Liu, Wei Wang, Xire Aili, Qian-ru Wang, Fan Xu, Hai-Xia Luo, Yan-Bin Shi, Ling-Ling Zhao, Hong-Jun Li

**Affiliations:** 1 Henan Clinical Research Center of Infectious Diseases (AIDS), Affiliated Infectious Diseases Hospital of Zhengzhou University, Henan Infectious Diseases Hospital, Zhengzhou, Henan, China; 2 Department of Radiology, Beijing Tiantan Hospital, Capital Medical University, Beijing, China; 3 Department of Radiology, Qilu Hospital of Shandong University, Jinan, China; 4 Echocardiography Medical Center, Beijing Anzhen Hospital, Capital Medical University, Beijing, China; 5 Department of Radiology, Beijing YouAn Hospital, Capital Medical University, Beijing, China; 6 Medical Imaging Department of Henan Infectious Disease Hospital, Zhengzhou, Henan, China; 7 Beijing Advanced Innovation Centre for Biomedical Engineering, Beihang University, Beijing, China; 8 Laboratory for Clinical Medicine, Capital Medical University, Beijing, China

**Keywords:** biomarkers, hand, HIV, immunity, neuroimaging

## Abstract

**Introduction:**

Combination antiretroviral therapy effectively controls viral replication in HIV-infected individuals, yet cognitive and motor impairments persist in 30–60% of patients, contributing to HIV-associated neurocognitive disorders (HAND). Current diagnostic approaches, such as the Frascati criteria, remain limited by practicality and sensitivity. Integrating neuroimaging with immunological markers may enhance early detection and mechanistic understanding.

**Methods:**

We conducted a literature search in PubMed and Web of Science for articles published between 1990 and 2024, using keywords including “HIV-associated neurocognitive disorders,” “HIV,” “immunity,” “neuroimaging,” and “treatment.” Additional relevant publications were identified through manual review of reference lists.

**Results:**

This review synthesizes current epidemiology, diagnostic methods, and neuroimmune mechanisms underlying HAND. It summarizes available neuroimaging techniques and associated biomarkers, emphasizing progress in combining neuroimaging with immunology to identify structural and functional neural changes. Abnormal neuroimaging findings coupled with immunological markers show promise for earlier detection compared to cognitive assessments alone.

**Discussion:**

We highlight the systemic connections between early HAND diagnosis and the synergistic use of neuroimaging and immunology, identifying candidate biomarkers in HAND pathogenesis. Furthermore, we discuss emerging treatment strategies and the relevance of early detection in the context of potential HIV eradication.

## Epidemiology and diagnosis of HAND

Since its first identification in 1981, the HIV/AIDS pandemic has affected approximately 84.2 million individuals, resulting in 40.1 million deaths linked to AIDS-related diseases. As outlined in the 2022 report by the Joint United Nations Programme on HIV/AIDS (UNAIDS), despite the availability of effective treatment methods and tools for preventing, detecting, and managing opportunistic infections, the global count of individuals living with HIV infections remained at approximately 38.4 million by the conclusion of 2021. Within the same timeframe, nearly 1.5 million new HIV infections were documented, along with 650,000 deaths attributed to HIV-associated conditions—equating to an average of one death per minute (1).

The HIV virus is neurotropic and can invade the central nervous system (CNS) early in the course of infection, leading to conditions such as encephalitis (2). Although antiretroviral therapy (ART) has achieved significant therapeutic success, HIV can persist in the CNS, resulting in various neurocognitive impairments and behavioral abnormalities, clinically referred to as HIV-associated neurocognitive disorders (HAND) (3). The clinical manifestations of HAND are primarily reflected in several key areas. First, some authors suggest that psychomotor slowing and mental slowing are the hallmark symptoms of HAND (4), and may even underlie unidentified deficits in neurocognitive domains (5). HIV-related motor slowing is commonly observed in tasks such as gait speed (6), finger tapping (7), and hand dexterity (e.g., grooved pegboard) (8). Slowed information processing appears in tasks both with (e.g., trail making) and without (e.g., Stroop color-word) motor demands. Additionally, others propose that the most common manifestations of HAND involve deficits in learning/memory and executive function domains (9, 10). Consistent with other neurocognitive domains, the severity of attention/working memory impairments in HAND patients seems to correlate with the severity of HIV disease and the complexity or “load” of attention/working memory tasks. In the early stages of HIV disease, basic attention/concentration skills appear relatively spared (11), but in later stages (e.g., CD4 < 200), HAND patients may exhibit mild to moderate deficits (11, 12). Moreover, episodic memory impairments are highly prevalent among HIV patients, with estimates ranging from 40 to 60% (7, 13). HIV is also associated with executive function impairments, particularly in the later stages of the disease (11). Subtle deficits in spatial cognition may also occur in HIV infection, though the underlying mechanisms remain unclear (14).

The clinical diagnosis of HAND presently hinges primarily on the Frascati criteria introduced in 2007 (3). The comprehensive assessment for HAND entails the evaluation of a minimum of five cognitive domains, encompassing attention, information processing, language, abstraction/executive function, sensorimotor skills, simple motor skills, complex perceptual-motor skills, or memory (including learning and recall). Diagnostic criteria for asymptomatic neurocognitive impairment (ANI) dictate that patients’ neuropsychological test scores should register at least one standard deviation (SD) below normative data in at least two cognitive domains, all the while maintaining unimpaired daily functioning. Parallelly, PLWH present commensurate neuropsychological test outcomes, yet, in the presence of compromised daily functioning, a diagnosis of mild neurocognitive disorder (MND) is aptly ascribed. Those afflicted by HIV-associated dementia (HAD) must exhibit pronounced deficit within a minimum of two functional domains, typically culminating below normative benchmarks, coupled with more pronounced degradation in activities of daily living (ADL). Despite the initial intent of these criteria to facilitate research endeavors, this nomenclature has traversed boundaries to guide the clinical allocation of disease burden (15). Notwithstanding, recent investigations have proffered compelling evidence that the Frascati criteria may impart a notable inflationary bias upon the prevalence estimation of HAND, thus underscoring the exigency of recalibrating the criteria to optimize the delineation of individuals at heightened risk of developing HAND (16).

Epidemiological investigations reveal that in the post combination cART era, the majority of PLWH experiencing HAND exhibit milder symptoms. Less than 5% of those diagnosed with HAND among PLWH suffer from HAD (9, 17, 18), a condition notably rare, A predominant presentation involves asymptomatic neurocognitive impairment (ANI) or MND (3, 16, 19–23). Furthermore, distinctions in the neuropathological pattern between the pre- and post-cART eras are evident: before cART, impairments were more pronounced in motor skills, cognitive speed, and speech fluency, whereas the cART era witnessed more substantial impairments in memory (learning) and executive functions (23). Despite cART’s improvement in viral suppression and immune reconstitution, it is evident that as the population of individuals with chronic HIV infection grows, cognitive impairments persist, negatively impacting the quality of life and daily activities of people living with HIV (22). HAND have emerged as a significant complication detrimentally affecting HIV-infected individuals in clinical settings. Strategies to mitigate the incidence of HAND, especially ANI, among HIV-infected individuals stand as crucial measures to enhance patient quality of life.

In light of these findings, it becomes imperative to ascertain whether novel biomarkers, neuroimaging techniques, and imaging markers can indeed enhance the identification of high-risk populations. Moreover, it is advisable to adopt a cautious approach when tracking the progression of neurodamage in individuals with ANI (24). Screening ANI patients warrants consideration, as PLWH affected by ANI exhibit poor treatment adherence and elevated unemployment rates (25), and are notably correlated with a heightened risk of progressive cognitive dysfunction (26).

## Immunological mechanisms of HAND

Given that the initial extracellular domain of the CD4 molecule serves as the binding site for the envelope protein gp120 of the HIV, the human CD4 molecule emerges as the principal receptor for HIV. Additionally, the chemokine receptors CXCR4 (CD184) and CCR5 (CD195) act as auxiliary receptors for HIV on T cells and monocytes. Recent research has harnessed the gene co-expression network modular analysis approach to dissect gene expression profiles from cases within the National NeuroAIDS Tissue Consortium (NNTC) in the United States. Notably, a negative enrichment of genes associated with immune response regulation was observed in the frontal cortex. These genes are presumed to be under the regulation of transcription factors IRF8 and SPI1, alongside Type I and Type II interferons. This finding serves as compelling evidence that immune response alterations contribute to the pathogenesis of HAND (27).

Currently, two major theories outline potential routes for HIV entry into the central nervous system (CNS). Firstly, the “free virus hypothesis” suggests that when blood–brain barrier dysfunction leads to increased permeability, the virus might directly infiltrate brain tissue. Secondly, the “Trojan horse hypothesis” posits that activated lymphocytes and monocytes infected with HIV traverse the blood–brain barrier, carrying the virus into the CNS. The translocation of monocytes within the blood–brain barrier stands as a pivotal step in the development of HAND. Most HIV viruses entering the brain do so within infected monocytes, which breach the blood–brain barrier to replenish the numbers of perivascular macrophages (21). In comparison to most monocytes (CD14highCD16-), CD16 + monocytes express higher levels of CCR5, rendering them more sensitive to HIV-1 entry, facilitating replication, and serving as a persistent viral source during HAART therapy (28). Even with successful antiretroviral therapy, 40–70% of HIV-infected individuals are impacted. Despite virological control, the frequency of peripheral blood CD14(+)CD16(+) and CD14(low)CD16(+) monocytes is elevated in HIV-seropositive patients compared to seronegative individuals (29). Further research even indicates that dopamine can enhance the translocation of CD14 + CD16 + monocytes across the blood–brain barrier into the central nervous system (30).

The brain acts as an enduring reservoir for the HIV, persistently harboring the virus even in patients undergoing effective combination therapy (31–33). Consequently, it can serve as a potential host for ongoing HIV infection, notably intertwined with sustained immune activation within the CNS (34, 35). Two principal processes are considered as the underlying mechanisms leading to neuronal damage in HAND: direct neurotoxicity caused by HIV and/or its viral proteins (Tat, gp120, Vpr) resulting in neuronal death, and more significantly, viral infiltration of microglia, astrocytes, and macrophages within brain parenchyma. Once activated and infected, these cells may release viral proteins, neurotoxins, cytokines, and chemokines. This intricate interplay of neuroimmune activation and neuroinflammation indirectly inflicts neuronal damage, known as the “bystander effect.” Initiators of the inflammatory pathways potentially driving neuronal apoptosis are likely TNF-*α* and IL-1 (30, 33, 36).

The notion proposed by Russell et al. (37) challenges the presence of HIV antigens within astrocytes of primary infection sites. An increasing body of evidence, however, points to brain macrophages and microglia as pivotal cellular contributors to HIV-1 infection within the CNS, carrying HIV DNA and mediating the neurodegeneration observed in HAD patients (21, 38). Microglia, categorized as CNS macrophages, serve as the primary cellular reservoir for concealed HIV-1 within the brain (39). Their activation induces neuroinflammation, hastens brain aging, and fosters HAND. Notably, [11C]DPA-713 PET imaging by Rubin et al. revealed an inverse correlation between microglial activation and cognitive function in HIV patients (40). However, the role of astrocytes, despite supporting only a restricted, non-productive infection, is now recognized as crucial in HAND pathogenesis. Astrocytes can be infected via CD4-independent mechanisms, potentially involving alternative surface molecules or trans-infection through contact with infected CD4 + cells (41, 42). Although they produce little progeny virus, infected astrocytes persistently express neurotoxic viral proteins like Tat and Nef, and their infection or subsequent apoptosis disrupts critical CNS homeostatic functions (e.g., glutamate regulation, BBB maintenance, microglial quiescence) (43). This dysfunction amplifies neuroinflammation and neuronal injury, acting synergistically with productive infection in microglia/macrophages. Therefore, the consensus has evolved to view microglia/macrophages as the primary productive reservoir, while astrocytes serve as a key pathological amplifier and contributor to the inflammatory cascade in HAND (44).

Moreover, multinucleated giant cells have been identified as hallmark neuropathology of HIV infection, resulting from fusion of infected and uninfected macrophages and microglia (21). These findings challenge the consensus of astrocytes as the primary HIV viral reservoir in the human brain (45). Therefore, targeting microglia is of paramount significance in treatment strategies, albeit a formidable endeavor (46). Nonetheless, even with low HIV infection rates in astrocytes, their impact remains noteworthy. Eliseo Eugenin’s work highlights that even a limited number of HIV-infected astrocytes can disrupt the integrity of gap junctions, leading to widespread blood–brain barrier impairment. This can further induce bystander damage in uninfected astrocytes, amplifying the toxic impact through a novel mechanism (47, 48).

## Cerebrospinal fluid and blood immunological biomarkers in HAND

CNS immune activation stands as a distinctive feature of primary HIV-1 infection. Regardless of viral load and the lowest/current CD4 + counts, significant differences in cerebrospinal fluid (CSF) immune biomarkers are evident between HIV-positive individuals, with or without accompanying neurocognitive dysfunction (49). Some cytokines can serve as assessment indicators for ongoing HIV infection (50), even in participants with the lowest cerebrospinal fluid viral loads (51). Immune activation may precede reliable viral detection (52). Consequently, the identification of efficient and accessible immunobiomarkers could aid in advancing our understanding of the neuro-pathology, diagnosis, and prognosis of HAND.

Heterogeneity exists in the dynamics of virus presence between plasma and CSF, with distinct regional differences observed between CSF virus and brain tissue virus post-infection (53). Plasma biomarkers, such as hemoglobin levels, platelet counts, and sCD163, offer advantages in terms of easy collection. Remarkably, HIV can be detected in CSF as early as Day 8 of infection, preceding plasma detection and emerging in brain tissue by Day 15 post-infection (54). CSF, synthesized within the ventricle choroid plexus, has been previously shown to reflect infiltrating brain parenchymal immune cells in other neuroinflammatory conditions (55, 56). As such, CSF may offer distinctive diagnostic insights for infectious, inflammatory, and neurodegenerative CNS disorders (57–59). As our ability to identify patients at increased risk of HAND using these biomarkers improves, our capacity to implement neuroprotective therapeutic strategies for optimal patient care will also grow. This section will elaborate on the progress and potential applications of research on potential immunological biomarkers of HIV in cerebrospinal fluid, plasma, and brain tissue.

### Immune cells

Increasing evidence suggests that immune cells play crucial roles in the pathogenesis of HAND. To date, four immune cell types have been implicated in supporting HIV invasion of the CNS. These cells include CD14^+^CD16^+^ monocytes, perivascular macrophages, CD4^+^ T cells, and CD4dim CD8bright T cells. These HIV-infected immune cells release the virus in the brain, support infection of microglia, and contribute to limited non-classical infection of astrocytes (60).

In perinatally HIV-1 infected adolescents and young adults undergoing ART, immune activation and exhaustion markers within T cell subsets have been shown to correlate with viral persistence (61). Irrespective of the pre-cART and cART eras, lower CD4 nadir values have been predictive of neurocognitive impairment (NCI) (23). A recent small-scale study involving participants with acute HIV Fiebig III/IV stages highlights the potential role of CD4^+^ T cells in early infection as carriers of HIV to the central nervous system (62). Levels of cell-associated viral RNA (CA-RNA) and DNA of HIV-1 within CSF were significantly higher per cell than those in PBMCs. Notably, the most plausible source of HIV-1 CA-RNA and DNA in CSF cells appears to stem from CXCR3^+^CCR5^+^CD4^+^ T cells transported from the peripheral blood (63).

In the context of HIV infection, CD8^+^ T cells emerge as a prominent cellular constituent within the CSF, exhibiting a significant increase when compared to uninfected subjects (64), this can only be partially reversed by ART. CD8^+^ T cells may participate in CNS immune surveillance by mediating the clearance of infected CD4^+^ T cells, monocytes/macrophages, and resident cells (65). Activated CD8^+^ T cell levels are elevated in individuals with HAND compared to those with normal cognition (NL), and they are directly correlated with plasma viral load while negatively associated with cognitive status. Additionally, diminished gag-specific cytolytic activity (CD107a/b^+^) has been observed in HAND patients when compared to NL individuals, and this reduced activity is linked to their neurological performance, suggesting the potential involvement of cytotoxic CD8^+^ T cells in the mechanisms underlying HAND (66). Even in individuals with well-controlled ART, extensive infiltration of CD8^+^ T cells into the brain can lead to CD8^+^ T cell-associated encephalitis and severe neurocognitive impairment (67).

In comparison to the bloodstream, PWH exhibit an increased frequency of dendritic cells and a decreased frequency of B cells and NK cells within the CSF. Despite undergoing antiretroviral therapy, the transcription of HIV-1 within central memory CD4^+^ T cells of PWH’s CSF remains ongoing in the central nervous system. The activation of TH1-mediated CD8^+^ T cells serves as a hallmark of CNS immune response during the chronic phase of HIV infection. These findings underscore the utility of multi-modal single-cell analysis in probing the central nervous system during chronic HIV infection (68).

The percentage of monocytes in the bloodstream correlates with the progression of HIV and the severity of CNS tissue pathology (53). In HAD, cerebrospinal fluid can exhibit monocytosis with up to 20 cells/L (69). Regression analysis and machine learning algorithms suggest that increased monocyte turnover is more strongly correlated and contributes to shortened survival time compared to decreased CD4^+^ T cell count, plasma viral load, or viral strain with reduced survival time (70). Similarly, Claudia R. Avalos has also confirmed in an animal model of SIV infection that potential macrophage reservoirs exist in the brains of macaques treated with ART. This supports the perspective that besides quiescent CD4^+^ T cells, other latent host cells exist and underscores the importance of macrophages in devising strategies for HIV eradication (71).

Compared to blood, CSF harbors a higher proportion of CD8^+^ T cells and two distinct myeloid cell subsets. Flow cytometry allows the discrimination of microglia from other myeloid populations present in CSF. In contrast to peripheral blood mononuclear cells (PBMCs), CSF exhibits an increased frequency of microglia-like cells (72, 73). Shelli F. Farhadian pioneered single-cell RNA sequencing (scRNA-seq) to comprehensively characterize the immune cell landscape within CSF at the transcriptional level. They uncovered a rare (<5% of cells) myeloid subset (circulating microglia-like cells) present exclusively in CSF, with gene expression features significantly overlapping those of microglia associated with neurodegenerative diseases (72). These subsets might perpetuate neuronal damage in the context of HIV infection. Moreover, their presence potentially connects to the chronic immune activation within the central nervous system during HIV infection.

### Cytokines

Inflammatory cytokines and chemokines amplify the trafficking of immune cells into the brain, consequently elevating HIV viral load and inducing immune responses in resident macrophages and microglia. Regardless of cognitive status, markers of immune activation (including sCD163, sCD14, neurofilament light chain (NFL), glutamate, neopterin, CCL2, CCL3, CXCL-10, IFNγ, high mobility group box 1 (HMGB1), IL-6, IL-8) remain elevated in the plasma/cerebrospinal fluid of ART-suppressed PWH patients (65, 67, 74–79), suggesting that continuous intrathecal inflammation and neuronal damage persist even in asymptomatic patients.

In summary, specific monocyte activation (elevated levels of neopterin, sCD163, sCD14) and neuroinflammatory markers (increased levels of IFN-*γ*, IL-1α, IL-7, IL-8, sTNFR-II, and decreased levels of IL-6) exhibit a consistent directionality associated with HIV-related neurocognitive impairment (49), Conversely, indicators such as CCL2, IL-6, and IFN-γ in the CSF of animals receiving ART show significant reductions (80).

#### Interleukin

In HIV + patients, pleocytosis leads to elevated levels of IL-2, IL-6, IL-7, IL-8, and IL-10 in the CSF (81). IL-6 is a molecule produced during infection and tissue damage. Its upregulation in plasma is associated with HIV infection regardless of whether HIV patients undergo ART treatment (82, 83), and it is independently correlated with the severity of HAND (84). The concentration of the pro-inflammatory cytokine IL-6 in the CSF of HAND patients is elevated and remains so even after 12 weeks of treatment (85). However, differing perspectives have been presented. Some studies suggest that in plasma, controlling for gender, NCI is associated with higher plasma IL-6 levels, but there is no correlation with CSF levels (86). There are even studies indicating that lower CSF levels of IL-6, IL-1β, IL-12, and IL-10, along with higher INF-*γ*, IL-4, and IL-7, are linked to poorer semantic fluency (87). This discrepancy may be attributed to variations in study populations, sample sizes, and the lack of immune control groups in some studies. Additionally, elevated plasma IL-6 is associated with older age, non-Black ethnicity, higher body mass index, lower lipid levels, HIV replication, lower CD4(+) cell counts, protease inhibitor use, comorbidities, and decreased eGFR (kidney function decline). This suggests that multiple factors influence HIV-related inflammation, warranting careful consideration when assessing IL-6 as a biomarker for clinical outcomes (82, 83). Elevated levels of IL-8 in the CSF are associated with HIV-related neurocognitive impairment, with no significant impact observed from HAART treatment (88). However, post-ART, the pro-inflammatory gene IL-9 remains significantly upregulated (80). Research also indicates that the increased IL-33 induced by HIV-1 infection in astrocytes and neuronal cells correlates with the differential manifestation of HIV neuropathogenic mechanisms, including induction of neuronal apoptosis, synaptic dysfunction, and neuroinflammation (89).

#### Interferon

Interferon-alpha (IFN-*α*) is primarily produced by monocytes/macrophages and lymphocytes. In the pre-combination cART era, elevated levels of IFN-α were observed in the CSF of late-stage HIV-associated dementia patients. During the cART era, IFN-α exhibited a negative correlation with various indicators of neurocognitive function, including composite scores reflecting overall performance, and showed close association with CSF NFL levels (90). Interferon-gamma (IFN-*γ*) is a pleiotropic cytokine primarily released by activated T cells and NK cells. Under normal circumstances, these cells do not pass through the blood–brain barrier at detectable levels, rendering them generally undetectable within the CNS (91). However, during responses to CNS infections such as during HAND, there is a significant increase in the trafficking of T cells across the blood–brain barrier. This results in elevated levels of CSF interferon-gamma (IFN-*γ*), which correlates with the severity of neurocognitive deficits in HIV + patients (81, 92). Elevated IFN-γ levels have been observed even in HIV + patients without clinical manifestations, suggesting its involvement in the perpetuation of HAND. Prolonged expression of IFN-*γ* has been shown to downregulate heme oxygenase-1 (HO-1) expression in human astrocytes (93). HO-1 is renowned for its neuroprotective properties and role in preventing oxidative stress-induced damage. This could partially explain why IFN-γ is considered a key driving factor in HAND pathogenesis.

#### Colony stimulating factor

Elevated levels of cytokines M-CSF and G-CSF in the CSF are associated with HIV-related neurocognitive impairments (88, 94). Furthermore, CSF1R expression remains consistently high in microglia and perivascular macrophages (PVM) within the brains of HIV-1 infected patients on suppressive ART. This sustained expression and activation are likely maintained by a self-sustaining inflammatory loop: the continuous presence of its ligand CSF1, potentially secreted by residual infected or activated brain macrophages, leads to persistent CSF1R phosphorylation and downstream STAT5 signaling. This process drives chronic microglial and PVM activation, resulting in the release of neurotoxic cytokines and aberrant synaptic pruning, thereby contributing to neuronal injury independently of active viral replication (95). In the SIV-infected Chinese-origin rhesus macaque model, there is a downregulation of the Colony-stimulating factor (CSF1) gene, possibly linked to the differentiation of macrophages and microglia (80). Conversely, another study reported an upregulation of CSF1 in the small microglia cells of the SIV RM model. Elevated CSF1 expression was closely associated with the upregulation of genes involved in antiviral and oxidative stress responses in microglia. However, this research was conducted in the frontal cortex of male juvenile pigtailed macaques (*Macaca nemestrina*) rather than in Chinese-origin rhesus macaques (chRMs), which might explain the discrepancies due to the different brain regions studied and species used (96). Subsequently, their team found that although both TREM2 and CSF1R are tightly correlated in terms of mRNA expression control and downstream functions in microglia, CSF1R might play a more significant role in the pathogenesis of HAND. CSF1 is primarily associated with chronic microglial activation and initiation, followed by acute microglial immune responses. CSF1R protein levels remain elevated even in monkeys where SIV replication is fully suppressed after initial infection. This could prepare the cells for subsequent damage (97), consistent with its role in sustaining the pathogenic cycle described above and preparing the cells for subsequent damage.

#### Chemokine

Monocyte chemoattractant protein-1 (MCP-1), also known as chemokine ligand 2 (CCL2), is a pro-inflammatory chemokine that enhances viral replication and pathogenesis. Research has revealed that both HIV and its viral products augment the expression of this chemokine in HIV patients. Even under ART, MCP-1 levels remain closely associated with observed immune activation, inflammation, and HIV-related neurocognitive impairment in patients (88, 98). In a rat model, bilateral hippocampal injection of CCL2 led to impaired spatial memory and cognition within 6 h, potentially through upregulated mRNA expression associated with three key pathological events: inflammation, excitotoxicity, and neuronal apoptosis (99). CCL2 plays a critical role in the migration of HIV + cells across the blood–brain barrier (100), recruiting a unique CCR2/5 + CD4 + T cell subset that, upon infection, establishes a significant reservoir for latent infection. CCL5, a natural ligand of the HIV entry receptor CCR5 in monocytes, recruits monocytes to inflammatory sites in the CNS. Elevated levels in HIV + patients without neurocognitive symptoms suggest an increased chemotactic effect on monocytes that could contribute to the inflammatory state of the central nervous system (101).

CXCL10, which primarily recruits monocytes and T cells to inflammatory sites. Beyond its chemotactic properties, CXCL10 has been implicated in inducing neurodegeneration through CNS calcium dysregulation. In the context of HIV infection, CXCL10 produced by Th1 cells is associated with HIV replication, and elevated plasma levels of this chemokine during the early stages of infection serve as markers for rapid progression of AIDS (102). Exploring the relationship between cognitive function biomarkers and gender, CXCL10 has been identified as a specific biomarker for NCI in chronically HIV-infected females (86). Building upon this, combined plasma levels of CXCL9, CXCL10, and CXCL11 in primary HIV-1 infection (PHI) patients have been shown to predict the long-term prognosis of HIV disease in the men who have sex with men (MSM) population, potentially emerging as novel clinical biomarkers (103). Small glial cells or macrophages infected with HIV-1 regulate CXCL12 production in astrocytes through IL-1β, providing ligands for upregulated CXCR4, a mechanism related to the pathogenesis of HIV-related brain diseases (80, 104). Signaling from gp120 to CXCR4 is believed to activate multiple pathways that converge directly or indirectly toward neuronal injury (105).

IP-10, MIP-1β, and MCP-4 are C-C chemokines that target monocytes and macrophages. Both MIP-1β and IP-10 induce pro-inflammatory genes in microglia cells (106). In the brain, IP-10 has been shown to attract activated T lymphocytes and monocytes by binding to CXCR3 chemokine receptors and, to some extent, binding to CCR5 chemokine receptors (107). IP-10 is associated with HIV replication in monocyte-derived macrophages, contributing to the accumulation of activated T cells in the CSF compartment of HIV-1-infected individuals. Neutralizing IP-10 can reduce HIV replication in these cells (108). Elevated levels of the cytokine IP-10 in the CSF are correlated with HIV-associated neurocognitive impairment, even more so in cognitively impaired patients receiving HAART treatment (88, 109). MIP-1β is a natural ligand for the CCR5 chemokine receptor and serves to promote leukocyte accumulation while aiding in protective immunity against HIV-1 (110). Concentrations of the pro-inflammatory cytokine MIP-1β are elevated in the CSF of HAND patients, and these levels remain elevated even after 12 weeks of treatment (85). The impact of host genetic variations on susceptibility to HIV-1, with the primary focus attributed to genes encoding Human Leukocyte Antigen (HLA) class I and chemokine receptor CCR5, plays a significant role (111).

The chemokine fractalkine (FKN, CX3CL1) exists in both membrane-anchored and soluble isoforms, and it has been proposed to be involved in the onset and progression of inflammatory brain diseases. In the brain, this chemokine is predominantly expressed by neurons, while its receptor (CX3CR1) is primarily expressed by microglial cells, establishing an interplay between the two (112). Upon binding with its receptor CX3CR1, FKN induces leukocyte adhesion, chemotaxis, and activation, including brain macrophages and microglial cells. FKN expression is upregulated in the brain tissue and cerebrospinal fluid of individuals with HAD (113). However, there are also reports of downregulated FKN gene expression (80). The HIV-1 Tat protein suppresses CX3CR1 expression in microglial cells through the NF-κB-YY1 pathway, attenuating CX3CR1-mediated microglial functional responses, and consequently inducing pro-inflammatory alterations (114).

#### Other cytokines

In HIV-positive patients, elevated levels of TNF-*α* are observed in the CSF (81). In plasma, higher levels of TNF-α are associated with NCI (*p* < 0.05) after controlling for gender (86). In comparison to FGF-2, MCP-1, or neopterin, lower levels of FGF-1 are correlated with impairment in five out of seven cognitive domains (115). Clinically significant dysregulation of the insulin-like growth factor (IGF) family proteins occurs in individuals with HIV infection; however, no significant correlations were found between HAND and reduced levels of plasma IGF1, IGF2, or cerebrospinal fluid IGF1 (116).

### Leukocyte differentiation antigen

In individuals with suppressed viral load, plasma sCD163 levels can distinguish milder forms of HANDs, serving as a plasma biomarker for patient neurocognitive impairment (117, 118). Plasma sCD14 is associated with impaired neurocognitive performance in attention and learning domains of individuals with late-stage HIV infection, indicating the involvement of cortical and subcortical pathways in the era of cART (119). Even in patients receiving cART, elevated CSF sCD14 levels persist and correlate with neurocognitive test impairment, suggesting ongoing intrathecal inflammation even in the absence of clinical manifestations. This underscores its potential as a biomarker for monitoring HAND progression and a predictive factor (79, 120). A combination of three biomarkers (sCD14, MCP-1, SDF-1α) can be employed for the diagnosis and prognosis of NCI (120).

### Complement

The complement system is a critical regulatory factor of both innate and adaptive immunity, playing a significant role in combating various viral infections through the formation of membrane attack complexes or opsonization to recruit phagocytic cells (121). The complement system is linked to the neuropathogenesis of HAND. Elevated levels of Complement component 3 (C3) protein were observed in the frontal cortex (FC) tissue of HIV deceased individuals with cortical Aβ plaques and diagnosed HAND patients. Although changes in Complement factor H (CFH) protein levels aren’t directly associated with the overall status of HAND, they correlate with deficits in executive and motor function (122). However, Rozek et al. reported contradictory findings, indicating a downregulation of C3 in the CSF of HAND patients compared to HIV-1 infected individuals without HAND (123). This discrepancy could be attributed to CNS complement activation associated with HIV-1 infection. Alterations in C3 levels might reflect different stages of central nervous system disease progression in infected individuals. As the disease progresses, anti-HIV-1 antibodies can trigger complement activation in CNS cells, such as microglia and astrocytes, involved in HIV-1 infection. However, this complement activation does not lead to clearance or eradication of HIV-1 within infected cells (66).

The complement system can be activated by HIV-1 envelope protein, mannose-binding lectin (MBL), and anti-HIV-1 antibodies, resulting in both resistance and enhancement of HIV-1 infection. Additionally, HIV-1 can hijack complement regulatory factors such as CD59 and CD55, as well as utilize these regulators along with complement factor H to evade complement attack. In a normal scenario, brain complement levels are much lower than in plasma, and complement deposition within brain cells is minimal or absent. However, in the context of HIV-1 infection in the brain, local complement generation and deposition significantly increase, suggesting potential involvement of complement in HAND pathogenesis. Aberrant complement activation occurs in the brain during HIV-1 infection, while complement regulation systems are downregulated. This imbalance between complement activation and regulation may contribute to neuronal damage during HIV-1 infection (124). Expression of complement proteins C9, C5L2, C5aR, and C3aR is markedly elevated in HIV-transfected microglial cells (66). In a study from 2016, CSF C1q expression levels were found to be significantly increased in cognitively impaired participants aged 18–24 compared to cognitively normal individuals. Moreover, CSF C1q levels were correlated with CSF NFL levels (125). Ahmed et al. (126), utilizing state-of-the-art LC/MS-based proteomic technologies, identified proteins significantly upregulated in HAND related to inflammation and the classical complement system, including serum amyloid P-component (APCS), complement C1q subcomponent B subunit (C1QB), and complement C1s subcomponent (C1s). Proteins associated with immune response pathways that were significantly downregulated in HAND included programmed cell death ligand 2 (PDCD1LG2), nectin-1 (NECTIN1), macrophage colony-stimulating factor 1 receptor (CSF1R), and CD166 antigen (ALCAM).

### Immunoglobulins and antigens

During suppressive ART, proviral HIV-1 DNA can be detected in the brains of a significant subset of individuals despite systemic viral suppression, indicating the presence of a latent viral reservoir within the central nervous system (38). Similarly, in a Pigtailed macaques model, long-term ART-mediated viral suppression revealed the presence of latent SIV genomes within the central nervous system (127). Early initiation of ART was significantly associated with reduced molecular diversity of HIV DNA in the CSF (*p* < 0.05), and among 8 participants with available paired HIV DNA sequences, 6 exhibited compartmentalization between CSF and blood HIV DNA populations (128). Following the initiation of ART, the decline of HIV RNA in cerebrospinal fluid lags behind that in plasma (85). Prolonged detection of HIV RNA in cerebrospinal fluid, rather than plasma, is associated with an increased risk of new-onset depression in HIV patients (129). Elevated plasma viral RNA is considered a predictor of CNS-related diseases following SIV infection in macaques (130).

Simultaneously, various HIV viral proteins may influence the pathogenesis of HAND. For instance, the HIV protein Tat, a viral trans-activator, is generated by cells with active HIV replication and released into the extracellular space or transferred from latent HIV-infected cells to neighboring uninfected cells, triggering upregulation of inflammatory genes and pathway activation, leading to cellular toxicity, even in the presence of effective ART (131). Tat can also bind to the promoter of gap junction protein 43 (Cx43), mediating increased expression of Cx43. This sustains functional gap junction communication in human astrocytes, promoting the propagation of intracellular toxic signals (30, 132). Therefore, inhibiting the action of HIV Tat could potentially mitigate the destructive consequences of HIV infection within the CNS. In addition to Tat, gp120, released by infected cells, induces an outward potassium current in microglial cells, playing a crucial role in neurodegeneration (133). Nef, an auxiliary protein encoded by HIV, is another such protein with impact. PBMCs containing Nef and extracellular vesicles carrying Nef can adhere to endothelial cells, transferring Nef into the endothelial cells. This leads to the production of CCL2 and subsequently causes blood–brain barrier leakiness (134), resulting in the release of pro-inflammatory cytokines, chemokines, reactive oxygen species, glutamate, and HIV proteins (gp120 and Tat), ultimately culminating in the development of HAND (29, 35).

In comparison to cognitively unimpaired individuals, HAND patients exhibit a higher coordination between Fc receptor (FcRn) binding, antibody titers, and functionality in their CSF, indicating an inflammatory shift in the antibody repertoire within the brain of cognitively impaired individuals. However, antibody titers and Fc-effector functions do not correlate with the severity of HAND, suggesting that antibody-mediated innate immune functions are unlikely to be the primary factors driving HAND pathogenesis (135). Total protein and albumin levels may show mild elevation due to blood–brain barrier disruption. Furthermore, the presence of oligoclonal bands and an elevated IgG index may be observed. However, these findings are nonspecific and frequently occur even during the asymptomatic HIV stage (136).

### Other immune activation and neural injury biomarkers

Neurofilament light chain (NFL) constitutes a major structural component of myelinated axons, playing a crucial role in maintaining axonal diameter and facilitating effective nerve conduction (137). Levels of NfL in CSF and blood are proportionally elevated in various neurological disorders, including inflammatory, neurodegenerative, traumatic, and cerebrovascular diseases, correlating with the extent of axonal damage (138). It serves as a sensitive yet nonspecific biomarker for large-caliber myelinated axonal injury (139, 140). A recent study has highlighted a close correlation between plasma NFL levels and CSF NFL, rendering it an exceptionally attractive candidate for early diagnosis of HAND (141). Elevated plasma NFL concentrations are significantly associated with poorer neurocognitive performance and show a pronounced decline following the initiation of combination cART (142). Notably, high levels of CSF NFL have been observed not only in HIV-associated dementia patients (143), but also in neurologically asymptomatic individuals with low CD4 counts, suggesting the presence of chronic neuronal injury even in this asymptomatic population (140). In the context of untreated acute HIV infection or initiation of immediate cART within 6 months, CSF NFL levels do not increase. However, in chronic HIV infection before and after treatment, CSF NFL levels display abnormalities (144). Although treatment reduces NFL levels, they still remain higher than in the control group, indicating either persistent virus-related damage or the aging effects of HIV infection (140). Furthermore, CSF NFL levels show a significant positive correlation with plasma HIV-1 RNA viral load and a significant negative correlation with plasma CD4 + T lymphocyte counts, suggesting a connection between neuronal damage and systemic HIV infection. CSF NFL also correlates positively with CSF pNFH, sCD163, and sCD14, underscoring the direct link between monocyte activation within the CNS compartment and neuronal damage across different stages of HAND (145). The association of NFL with neopterin and the albumin ratio suggests interrelations among axonal injury, neuroinflammation, and blood–brain barrier permeability (140).

Mild forms of HAND (ANI and MND) are associated with increased intrathecal immune activation. Neopterin serves as a biomarker to assess intrathecal immune activation in the CNS (34), and it is also indicative of macrophage and microglial cell activation. During early HIV-1 infection, untreated subjects show a progressive rise in neopterin levels in CSF along with the percentage of activated CD4 + and CD8 + T cells (146), suggesting an escalation of intrathecal inflammation, a prominent factor in HIV-associated neuropathology. Following viral suppressive ART, neopterin levels remain consistently abnormal in 41% of non-HAD patients and most HAD patients (147). Initiating ART during the chronic phase of HIV infection, even with elevated CD4 + T cell counts, does not correlate the occurrence of residual CNS immune activation (as indicated by cerebrospinal fluid neopterin concentration) with the pre-treatment immune state. This suggests that once the CNS reservoir is established, the timing of ART initiation during the chronic infection phase does not impact the CNS reservoir differently (148).

In addition to that, cerebrospinal fluid extracellular vesicles are elevated compared to HIV-negative controls (*p* < 0.05) and further increased in those with NCI compared to those without impairment (*p* < 0.05), showing a positive correlation with neurofilament light chain protein (NFL) levels (*p* < 0.001). Notably, in the cerebrospinal fluid extracellular vesicles of HIV-positive NCI patients, there is an enrichment of HLA-DR (*p* < 0.05), suggesting myeloid cells as potential sources of cerebrospinal fluid extracellular vesicles during HIV infection. Among HIV-positive individuals with neurocognitive impairment receiving cART treatment, the increase in CSF extracellular vesicles is correlated with the neuronal damage biomarker NFL, suggesting their potential as novel biomarkers for CNS damage (143). Moreover, in plasma from HIV-infected individuals, elevated levels of high-mobility group box 1 (HMGB1), NFL, and beta-amyloid protein in neuron-derived exosomes (NDEs) can distinguish cognitive impairment, indicating that NDE content might provide “real-time” reflection of neuronal health status, potentially aiding in understanding cognitive impairment and treatment strategies (149).

Neurological damage markers in HAND patients also include *β*-amyloid protein deposition and tau protein (30, 78). HIV can disrupt normal amyloid metabolism through the action of Tat, leading to the accumulation of Aβ protein. The tissue deposition of β-amyloid protein—associated with neurotoxicity, synaptic dysfunction, and memory impairment (149)—is likewise implicated in HAND, reflecting increased brain accumulation akin to that seen in mild Alzheimer’s disease (AD) patients (117, 150). Differentiating between them can be achieved by measuring CSF tau and phosphorylated tau (p-tau181) levels, which are either normal or mildly reduced (150, 151). More detailed investigations have shown that higher CSF T-tau and p-tau levels are associated with poorer neurocognitive performance in HIV-positive patients (152). Despite effective antiretroviral therapy, some HIV-positive patients still exhibit blood–brain barrier damage. The CSF/plasma albumin ratio (a marker of blood–brain barrier disruption) is significantly correlated with neuronal damage markers (total tau protein, phosphorylated tau protein, and A*β*-42), but not with cerebrospinal fluid neurofilament light chain protein concentration (153). Furthermore, autopsy of 43 HIV patients indicated potential neuro-pathological immunohistochemical markers for HAND, including synaptophysin (SYP) and microtubule-associated protein 2 (MAP2), as well as abnormal protein aggregates like β-amyloid protein, and markers for microglial/macrophage activation, astrocyte activation, and cytokine dysregulation (154).

Although NCI patients have higher levels of CSF NFL and S100B compared to HIV-infected individuals without impairment (*p* < 0.05), in adjusted models, only NFL is associated with NCI (143). The percentage of CD38+/HLA-DR + (CD8+) cells is independently correlated with more severe HAND (84). Additionally, alterations in circulating monocyte inflammasome components across different severities of HAND suggest that NLRP3 might serve as a potential biomarker or target to better understand and treat the link between systemic inflammation and HIV-related neurocognitive impairment (155).

Galectin-9 (Gal-9) is a soluble lectin with immunomodulatory properties that increases in plasma during HIV infection and induces HIV transcription. Analysis of Gal-9 tissue expression in postmortem brain specimens from HIV patients revealed its potential as a novel neuroimmune regulatory protein, possibly contributing to driving cognitive deficits in individuals with HIV infection and holding promise as a valuable marker for tracking cognitive abnormalities (156).

Recent investigations have also unveiled shed PrP(c) (non-pathogenic human prion protein) as a potential biomarker for neuronal damage (75). CCL2 and TNFα can stimulate the active conformation of metalloproteinase ADAM10, enabling it to cleave PrPc (75, 77). Comparing individuals with and without neurocognitive impairment, PLWH patients with neurocognitive impairment exhibit elevated levels of shed PrP(c) in their cerebrospinal fluid, believed to play a role in monocyte recruitment to the brain (75, 76). Shed PrP(c) leads to increased secretion of CCL2, CXCL12, and IL8 by astroglial cells, thereby exacerbating immune activation and neuronal damage (75).

While current research indicates the presence of numerous immune markers associated with HAND, recent studies have suggested that the diagnostic accuracy of plasma cytokine biomarkers’ cutoff values for defining neurocognitive impairment in HIV-infected individuals is only moderate to low (with IL-2 showing the highest sensitivity and specificity at 67 and 52%, respectively) (157). Incorporating a variety of cerebrospinal fluid and/or blood biomarkers, rather than relying on a single protein, may offer a more sensitive diagnostic biomarker profile for HAND (158). Therefore, the development of multimodal techniques is urgently needed for the early identification and clinical intervention of HAND.

## Neuroimaging biomakers

Neuroimaging stands as a pivotal tool for gaining insights into HIV neuropathology, offering diverse perspectives on the structural, functional, and molecular alterations induced by HIV (159). In the first 100 days following primary infection, structural brain changes, even in the absence of symptoms, can sometimes be discerned through neuroimaging (160, 161). With advancing age, neuroimaging changes in HIV patients become particularly prominent, including brain atrophy, abnormal white matter signals on MRI (especially in the basal ganglia region), and alterations in neural vascular coupling, as indicated by changes in resting-state and activation-induced BOLD signals in fMRI (151, 162).

Research has indicated differences in brain structure and function between HIV-positive individuals on long-term cART and HIV-negative individuals (163). However, James H. Cole and colleagues conducted a two-year longitudinal assessment of middle-aged PLWH who were successfully treated, utilizing comprehensive neuropsychological evaluations and multimodal neuroimaging techniques (including T1-weighted, T2-weighted, diffusion-weighted imaging [DTI], resting-state functional MRI, spectroscopy, and arterial spin labeling). They found that there was no increased risk of accelerated aging-related brain changes or cognitive decline in people living with the human immunodeficiency virus undergoing treatment (164). Consequently, the crucial issue of early diagnosis and timely intervention remains to be addressed in clinical practice.

### CT

**Figure 1:**
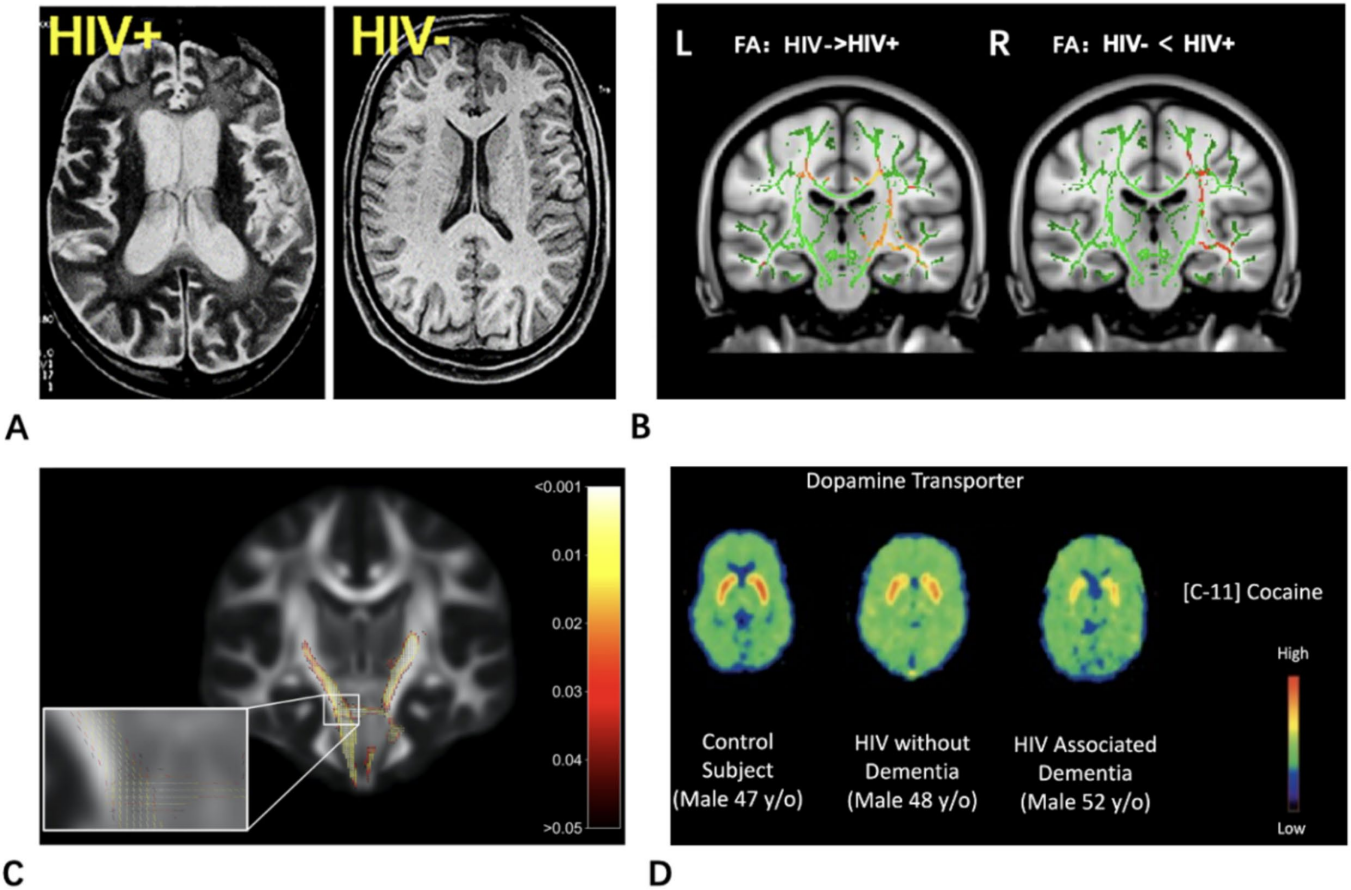
Typical neuroimaging findings in HIV^+^ patients. **(A)** 40-year-old HIV + individual showing the typical enlarged sulci and ventricles on T2-weighted magnetic resonance imaging (MRI) compared to the 38-year old HIV– individual with much smaller ventricles and sulci (shown on a T1-weighted MRI). Modified from Chang and Shukla (165). **(B)** Voxelwise comparisons for fractional anisotropy (FA) (L) and mean diffusivity (MD) (R) between HIV − controls and HIV + patients using Tract-Based Spatial Statistics (TBSS). Red: *p* = 0.05; Orange: *p* = 0.03; Yellow: *p* = 0.01. Adapted from Masters and Ances (177). **(C)** Coronal slice showing fixels that were significantly decreased (FWE-corrected *p*-values) in HIV^+^ individuals compared to HIV^−^. The zoomed-in area illustrates differences and *p*-values assigned to individual fixels in regions with crossing-fibers, around the cerebral peduncles (CP) and middle cerebellar peduncles (MCP). Fixels are colored by FWE-corrected *p*-value. Adapted from Finkelstein et al. (242). **(D)** A significant decrease in DAT availability was observed in the putamen and ventral striatum of HIV-positive patients with HIV-associated dementia compared with HIV-negative controls. Adapted from Wang et al. (216).

Plain or enhanced head CT scans have limited utility in assessing mild to moderate HAND but can provide some auxiliary diagnostic value in cases of irreversible HAD. In HAD patients, CT scans typically reveal diffuse, symmetric brain atrophy that is disproportionate to age, along with slightly decreased density in the corona radiata and centrum semiovale (165) (Figure 1A). It is important to note that these findings are non-specific and can be seen in other neurodegenerative or vascular conditions. Thus, their diagnostic value is primarily supportive, serving as objective evidence of substantial brain injury only within the confirmed clinical context of HAD. Additionally, CT can be used to rule out other opportunistic central nervous system lesions associated with AIDS.

### MRI

#### sMRI

Structural magnetic resonance imaging (sMRI) is employed to measure macroscopic differences in PLWH. Conventional T1-weighted MR imaging of macroscopic neuroanatomy can quantify global or regional gray matter (GM), white matter (WM), and cerebrospinal fluid. Brain atlases in standardized anatomical spaces enable automated segmentation of brain structures (166).

Research indicates that individuals with primary HIV infection (i.e., those infected within a year) exhibit reduced brain volume. Ragin et al. reported smaller gray matter volumes in PWH patients with an average duration of infection of 1 year (167). In subsequent analyses, Ragin et al. also found that individuals infected with HIV for less than 100 days had smaller whole-brain and gray matter volumes compared to people without HIV (PWOH) (160). In comparison to PWOH, individuals with a median duration of infection of 3.5 months had smaller caudate volumes (168). Israel et al. (169) using the colocalization-likelihood estimation (CLE) technique (a novel meta-analysis technique), quantified GM atrophy in HIV + adults. They discovered that GM atrophy in HIV + adults is primarily driven by two distinct but non-exclusive features: atrophy in the frontal lobe, including the anterior cingulate cortex (ACC), which is associated with HIV disease and consistently distinguishes HIV + adults from HIV controls, and atrophy in the caudate/striatum associated with neurocognitive impairment.

However, there are also studies reporting no volume differences between individuals with primary infection (median duration of infection 3.7 months) and those without HIV (PWOH), and even finding larger basal ganglia volumes in primary infection PWOH compared to individuals with chronic HIV infection (median duration of infection 90 months) (170). Bolzenius et al. (171), who conducted high-resolution MRI scans on PWH patients during AHI, also found that, compared to PWOH, participants had larger volumes in the amygdala, caudate, putamen, pallidum, and thalamus during AHI. Examination of AHI subgroups based on Fiebig staging (172) revealed that individuals in Fiebig III-V stages had larger volumes in the nucleus accumbens and putamen compared to those in Fiebig I-II stages. Late-stage Fiebig participants also demonstrated larger regional brain volumes compared to PWOH. These findings suggest that there are variations in volumetric measurements during the course of the disease, and the processes of structural changes still require further exploration. While the mechanisms behind these results remain unclear, they indicate that alterations in brain structure occur early in the HIV infection process, with the most significant changes observed later in the acute infection window (approximately 24 days post-initial infection). Furthermore, the observed increase in volume during AHI, rather than the commonly seen decrease in chronic infection, suggests that changes in brain structure associated with HIV infection are fundamentally nonlinear.

cART has been shown to prevent subcortical atrophy and cortical thinning (170). However, research has also demonstrated that in PWH under viral suppression, there are still defects in GM volumes in transition cortices, including the frontal and parietal cortices, insula, and cingulate, as well as subcortical structures (including the basal ganglia, thalamus, and hippocampus), when compared to HIV-negative controls (173, 174). Furthermore, recent multicenter studies have found that HIV elite controllers (ECs) exhibit the lowest cognitive performance, cortical thickness, and Fractal Dimensionality (FD) compared to HIV-negative controls (HCs) and PWH. This finding challenges our traditional understanding and raises suspicion that it may be associated with their long-term HIV infection. However, the sample size remains small, and other confounding factors cannot be completely ruled out. These data suggest that long-term cognitive effects might be a significant consideration in this population when contemplating cART. Overall, greater FD, cortical thickness, and hippocampal volume are associated with better cognitive function, but FD shows a stronger correlation with cognitive function compared to traditional measures of thickness and volume. This suggests that FD may be a more sensitive biomarker for cognitive changes (175). Therefore, the timely application of cART is a crucial consideration, whether in ECs or PWH.

sMRI quantifies brain atrophy and structural changes, where atrophy in the frontal lobe and striatum correlates with cognitive decline, though findings are often confounded by age and comorbidities.

#### DTI

Diffusion Tensor Imaging (DTI) is an MRI technique that measures the diffusion of water in tissues, and it has been used in HIV research since 2001 (176). It allows for the visualization of the distribution and orientation of white matter tracts. When assessing the integrity of brain white matter structures, especially when detecting subtle white matter abnormalities, DTI technology is currently the most ideal method. DTI of WM microstructure provides information about fiber organization through fractional anisotropy (FA) and unrestricted water motility via mean diffusivity (MD) (177) (Figure 1B). FA reflects the directionality of water diffusion and is sensitive to the overall coherence and integrity of white matter tracts. MD represents the overall magnitude of water diffusion. Further decomposition of diffusion into axial (AD, parallel to axons) and radial (RD, perpendicular to axons) diffusivities provides more specific insights: RD increases are strongly linked to demyelination, while AD changes may relate to axonal integrity. Abnormalities in DTI parameters indicate tissue damage and HIV-related neuroinflammation. HIV infection is associated with reductions in gray matter (rather than white matter) volume and white matter microstructural damage, typically unrelated to gray matter (178).

Quantification of brain changes within 100 days after HIV infection using DTI revealed white matter integrity loss in the corpus callosum (CC) and diffusion alterations in the caudate nucleus. These findings were detected before seroconversion, possibly representing one of the earliest brain changes in HIV infection. The occurrence of neural damage may be associated with the initial viral invasion, transient early viremia, and a significant period of immune suppression before antibody response (160). However, studies have also suggested that individuals with primary HIV infection (PHI) who did not receive antiretroviral therapy showed relatively preserved white matter microstructural integrity around a median of 4.1 months post-infection. This preservation was linked to blood–brain barrier disruption (as indicated by CSF/plasma albumin ratio and CSF protein) and a negative correlation between DTI metrics and estimated days of infection (179).

Specifically, HIV + patients exhibit diffuse white matter structural changes characterized by widespread reductions in FA and increases in MD compared to HIV-negative control groups (178, 180–182). This pattern (↓FA, ↑MD, ↑RD) suggests widespread microstructural disorganization with a significant demyelinating component. These white matter structural alterations are associated with CD4 cell counts below 500 cells/μL. While some studies have reported associations between white matter structural changes and HAND (178, 180, 182–184), a range of studies have also reported no such associations (173, 181, 185), possibly due to the successful treatment of HIV + patients and the elimination of other confounding factors. As the disease progresses to HAND, patients exhibit reduced FA in the right frontal lobe white matter, increased MD and RD in bilateral frontoparietal white matter, corpus callosum, bilateral corticospinal tracts, and bilateral cerebellar peduncles. These findings suggest that changes in frontal lobe white matter integrity contribute to cognitive impairment in HIV patients (184, 186). HIV infection has a greater impact on RD than on axial diffusivity (AD), indicating demyelination as a prominent pathology in white matter progression (184). Age exacerbates HIV-associated abnormalities of whole-brain white matter hyperintensities (WMH) and fronto-subcortical white matter integrity (187). Initiating cART can reduce neuroinflammation and improve DTI metrics (185), while perinatally HIV-infected children starting ART after the second year of life show associations between white matter changes on neuroimaging DTI and neuronal development (188).

DTI detects white matter microstructural damage, indicated by decreased fractional anisotropy (FA) and increased mean (MD) and radial diffusivity (RD), with elevated RD being more specific to demyelination and making the technique valuable for early monitoring.

#### BOLD-fMRI

Functional magnetic resonance imaging (fMRI) utilizing blood oxygenation level-dependent (BOLD) contrast can assess brain function during rest or task performance by measuring changes in the MRI signal associated with varying levels of oxygenated and deoxygenated hemoglobin. In functional MRI studies investigating the impact of HIV on the brain, meta-analyses have qualitatively linked dysfunction in the fronto-striatal circuit to cognitive impairment, disease progression, and treatment outcomes (189).

Resting-state functional MRI (rsfMRI) images are easily obtainable and amenable to various analytical approaches. The resting brain exhibits low-frequency spontaneous fluctuations, showing coherent activity across different spatial networks, which are utilized to estimate functional connectivity between regions. HIV infection leads to the disruption of resting-state correlations both within and between specific networks. Studies utilizing rsfMRI have revealed alterations in local intrinsic activity across different cognitive states in HIV-infected individuals. Among HAND patients, damage to the orbitofrontal cortex and primary sensorimotor areas is more severe relative to HIV patients with intact cognition (HIV-IC), and these changes correlate with behavioral performance, suggesting the involvement of these regions in HIV-related cognitive impairment (190). The impacts of HIV and aging are independent of each other (191), implying that HIV may accelerate brain aging and potentially increase susceptibility to neurodegenerative diseases. Active HAND in virally suppressed patients is significantly associated with reduced connectivity in salience and executive networks, revealing that HIV infection might expedite brain aging (192). However, there are also studies reporting negative results, such as Marloes et al., who found that HIV does not affect subcortical connectivity in virally controlled patients following cART treatment (193).

In contrast, task-related fMRI necessitates participants to perform specific tasks or view images that induce local brain activity changes. PWH typically exhibit lower task-induced activations in normal networks but show greater activations in reserve brain regions when faced with more demanding tasks and increased attentional loads (194–197), or risk-taking behaviors (198, 199). These studies suggest that neural abnormalities in PWH are associated with heightened cognitive demands. While implementing task-based fMRI in routine clinical care poses more challenges, it often involves larger changes in BOLD contrast, making it potentially more sensitive than rsfMRI in monitoring treatment or intervention effects (200).

rsfMRI reveal abnormalities in brain network connectivity and compensatory neural activation, with reduced functional connectivity being associated with cognitive impairment.

#### MRS

Magnetic Resonance Spectroscopy (MRS) stands as the only non-invasive technique capable of determining the chemical composition of specific tissue regions *in vivo*, allowing for the repetitive detection of HIV-related neuroinflammation and neural damage. Crucially, key MRS metabolites reflect distinct cellular processes: N-Acetylaspartate (NAA) is a marker of neuronal integrity; elevated Choline (Cho) indicates increased membrane turnover linked to inflammation or demyelination; and elevated myo-Inositol (mI) reflects astrocytic activation (astrogliosis). Furthermore, the concentration of metabolites measured by MRS can serve as a quantitative indicator of HIV-related brain injury, thereby offering opportunities to assess the effectiveness of cART in HAND (201).

A multicenter study revealed distinct alterations in HAND, characterized by increased abnormal white matter (aWM), inflammation, reduced gray matter volume, and lower NAA levels (202). Meta-analysis incorporating proton MRS data from 1993 to 2019 provided Class II evidence for neuro-metabolic differences during chronic HIV infection, associating it with lower total N-Acetylaspartate /total Creatine (tNAA/tCr), higher total Choline (tCho)/tCr, and higher myo-Inositol (mI)/tCr ratios. Elevated WM tCho/tCr and mI/tCr may potentially reflect reactive gliosis or myelin turnover. Neuro-metabolite measurements reliably detect the impact of chronic HIV infection and could contribute to understanding the pathophysiology of cognitive and sensorimotor decline following HIV infection (203).

The basal ganglia (BG) is a major affected region during early infection. Additionally, HAND patients exhibit lower glutamate (GLU) levels in the frontal gray matter, which may be attributed to reduced GLU reuptake by astrocytes, secondary excitotoxicity, and mitochondrial toxicity induced by antiretroviral therapy. The glutamatergic system likely plays a crucial role in the pathophysiology of HAND, and (1)H-MRS brain GLU may serve as an early surrogate marker for monitoring disease severity and treatment efficacy (204). In the era of cART, neurocognitive impairment early on manifests as increased Choline (Cho) and Myo-Inositol (Mi) in the frontal white matter and basal ganglia, followed by neuronal integrity loss resulting in N-Acetylaspartate (NAA) reduction later on (201). cART has been successful in partially controlling neuroinflammation, primarily reflected in the normalization of total myo-inositol (mI) and Cho, with fewer increases. However, neuronal dysfunction (NAA reduction) and neuroinflammation (increased mI and Cho) persist, leading to cognitive impairment in chronic PWH (205), concomitant with elevated immune activation markers (206). In perinatally HIV-infected children on cART, an increase in Cho/creatine (Cre) within the brain white matter compared to healthy controls is associated with poorer cognitive abilities, indicating continued glial cell proliferation, although no correlation exists between neuro-metabolites and neuronal injury markers in blood or CSF (207). Metabolic changes may take as long as 6–12 months to correlate with neuropsychological improvements, underscoring that cellular-level alterations require an extended period to manifest clinically, despite apparent clinical amelioration, and also indicating a longer duration for recovery (201). In a 5-year follow-up study of PWH under cART, progressive elevation in NAA/Cr was observed in the context of chronic HIV-related neuronal injury, followed by improved neurocognitive performance, potentially serving as an indicator of brain plasticity (208).

Magnetic Resonance Spectroscopy (MRS) has been extensively studied for HAD at a magnetic field strength of 1.5 Tesla. Higher magnetic field strengths, such as 3 Tesla, enable more reliable measurement of certain compounds, such as glutamate (Glu) and glutamine (Gln). 3T MRS measurements of Glx (the sum of Glu and Gln) may serve as a valuable marker for neuronal loss and dysfunction in HIV-infected patients (HAD patients exhibit reduced Glx concentration and Glx/Cr ratio in the frontal white matter, correlating with impaired specific cognitive domains like executive function, fine motor skills, attention, and working memory) (209). Glx and GABA appear to be more sensitive for identifying early neurocognitive impairment and treatment response (201), offering researchers in the field additional avenues for MRS exploration in the future.

MRS provides neurochemical profiles in which decreased N-acetylaspartate (NAA) indicates neuronal injury, while elevated choline (Cho) and myo-inositol (MI) suggest neuroinflammation and gliosis, making it useful for evaluating treatment response.

### PET

Neuro-HIV PET molecular imaging capabilities serve as a crucial complement to structural imaging. In the post-ART era, PET imaging has been employed to gain deeper insights into glucose metabolism disturbances, neuroinflammation, neurotransmitter system function, as well as amyloid/tau protein deposition in the brains of HIV-infected patients and HIV animal models. The preclinical and translational findings from these studies offer novel perspectives on the intricate pathophysiological mechanisms underlying HAND (210).

[18F]-FDG PET enables the quantitative measurement of cerebral glucose metabolism, reflecting neuronal and synaptic activity. It can quantify regional differences in brain glucose metabolism, providing information about brain pattern distribution and activation. Changes observed in FDG-PET in the post-cART era include varying severities of mesial frontal reduction in the relative metabolic rate of glucose and subtle basal ganglia hypermetabolism (211). Increased metabolism in the frontal cortex after the initiation of ART suggests that cortical dysfunction is reversible, and with viral control and increased CD4 + cell counts, cortical dysfunction improves. Therefore, early initiation of treatment after HIV diagnosis and secondary control of inflammation are necessary measures to prevent neurological damage in people with HIV (212). In 18F-FDG PET monitoring of SIV-infected macaques after interruption and cessation of ART treatment, an increase in brain glucose metabolism was correlated with viral rebound within 1 month and was consistent with elevated cerebrospinal fluid proinflammatory cytokine levels, which may reflect neuroinflammation during viral rebound. Meanwhile, initiation of treatment did not lead to significant changes in brain metabolism, suggesting that HIV-induced neuroinflammation may take longer to resolve than the follow-up periods we allow (213). This underscores the importance of not only diagnosing and treating HIV infection early but also adhering to continuous long-term treatment.

Positron Emission Tomography (PET) imaging, by measuring the density of the 18-kDa translocator protein (TSPO), offers the quantitative assessment of neuroinflammation *in vivo*. Increased binding of radioligands to TSPO provides an alternative indicator that can be used with PET to evaluate microglial cell activation in the brain. Jaime H. Vera and colleagues discovered that individuals with HIV who were effectively treated with ART exhibited an overall increase in the expression of TSPO. The areas with the highest uptake and neuroinflammation were found in the subcortical gray matter of the brain, particularly in the basal ganglia (caudate nucleus, putamen, and globus pallidus). Additionally, TSPO binding in the hippocampus, amygdala, and thalamus was associated with poorer overall cognitive performance in assessments of language and visual memory tasks. These findings suggest that microglial cell activation has a significant impact on both the functional and structural aspects of the brains of HIV-positive individuals undergoing treatment (214).

[11C]-cocaine is utilized to assess the availability of presynaptic dopamine transporter (DAT), while [11C]-raclopride measures the availability of D2 dopamine receptors, primarily reflecting postsynaptic sites. Studies have revealed diminished dopaminergic function, indicated by lower DAT availability, in HIV-positive individuals with cognitive impairment, which is associated with poorer cognitive performance. Meanwhile, alterations in dopamine (DA) D2 receptors appear to be insignificant (215, 216) (Figure 1D).

Currently, the majority of studies still employ the first-generation TSPO radioligand [11C]-PK11195. Different studies yield varying conclusions regarding whether there are differences in [11C]-PK11195 binding between HIV-positive individuals with and without cognitive impairment (217). These disparities in interpretation may stem from the relatively low signal generated by specific binding of [11C]-PK11195, making it challenging to accurately measure the brain-to-ligand binding. PET tracers such as FDG, C-pib, [C]-R-PK11195, and SPECT tracers like Tc-HMPAO, I-FP-CIT, and I-IBZM have been employed for HAND diagnosis, distinguishing dementia from non-dementia HIV patients, HAND from non-HIV-related dementia, and evaluating the impact of concurrent infections with other viral pathogens on brain function. Despite some intriguing findings, these tracers are not tailor-made for HAND and are not recommended for its diagnosis. To enhance HAND diagnosis and treatment, the development of specialized tracers is essential. Theranostic nuclear medicine might also emerge as a potential component of HIV therapeutic strategies (218).

PET enables the in-vivo detection of molecular-level processes, including neuroinflammation (via TSPO ligands), metabolic disturbances (FDG), and dopaminergic system dysfunction.

### Multimodality imaging

As brain imaging becomes increasingly prevalent in HIV care, and given the substantial interplay between brain structure and function, multimodal fusion holds the promise of providing neurobiomarkers for the diagnosis and treatment of cognitive impairments, thereby enhancing our understanding of the physiological mechanisms underlying HAND.

The reduction in gray matter volume in the left posterior cingulate gyrus of HIV-infected individuals aligns spatially with abnormal MEG responses (219). Combining high-resolution sMRI and MEG imaging to investigate neural oscillatory activity in the somatosensory system, people with HIV exhibit abnormally strong spontaneous cortical activity in the left postcentral gyrus, and this increased activity is driven by a local reduction in cortical gray matter thickness (220). By integrating three-dimensional T1-weighted imaging and resting-state functional imaging (combining VBM and rsFC), Liu et al. explored structural and functional alterations in PLWH, supporting the presence of brain atrophy and functional reorganization that correlate with neurocognitive function (221). Combining baseline measurements of DTI and 1H-MRS with sMRI volumes yielded the highest classification performance for cognitive outcomes in perinatal acquired HIV (CPHIV) children, incorporating 22 DTI and sMRI volume features (222). Individuals with cognitive impairment exhibited lower corpus callosum body (CCb) FA values and higher CCb functional connectivity, elucidating the functional relevance of the corpus callosum in HIV patients and providing a framework for understanding brain functional abnormalities in the context of structural brain anomalies, which may all contribute to cognitive impairment (223). Towgood et al. pioneered the combination of ASL and PET and found a consistent reduction in regional cerebral blood flow (rCBF) and regional cerebral glucose uptake (rCMRglc) in the anterior cingulate cortex (ACC) of HIV patients. Although the magnitude is small, this finding holds potential clinical significance (224).

In 2021, an analysis of PWH combined T1-weighted MR imaging, DTI, and rsfMRI to discover that lower cognitive function scores correlated with morphometric abnormalities. Simultaneously, shared alterations in brain structure and function involving compromised white matter integrity, abnormal activity in frontal, parietal, and occipital networks were identified, distinguishing HIV-infected individuals from non-HIV-infected individuals, and potentially contributing to cognitive impairment (225). Furthermore, studies introduced a Connectome-based Predictive Modeling (CPM) approach, where the integration of multimodal resting-state functional connectivity (FC) and structural connectivity (SC) features improved the accuracy of predicting cognitive scores in PLWH. The inclusion of clinical and demographic metrics may further enhance prediction accuracy by introducing complementary information, facilitating a better assessment of cognitive performance at the individual level (226).

At the same time, various risk factors also influence the cognitive function and neuroimaging (brain microstructure) changes in HIV patients. Risk factors for HAND in the cART era primarily include cardiovascular factors, age, hepatitis C virus infection, as well as other substance abuse (particularly methamphetamine), the lowest CD4 T-cell nadir, illicit drug use, risk factors for cerebrovascular diseases (diabetes, hypertension, hypercholesterolemia, obesity), and sleep disorders (insomnia, obstructive sleep apnea, sleep fragmentation), as well as comorbid mental conditions (severe depression, anxiety disorders, bipolar disorders). Additionally, research suggests that cognitive reserve, such as educational level, may also play a role (9).

Smoking is a significant contributing factor to brain atrophy and cognitive impairment in PWH (smoking can exacerbate or increase the risk of HIV-associated neurocognitive disorders). Although clinical studies consistently demonstrate the harmful effects of smoking, and nicotine has neurotoxic properties in the developing brain, preclinical studies on nicotine suggest mild cognitive enhancement, neuroprotection, and potential anti-inflammatory effects (227). PWH who smoke exhibit greater brain atrophy (both overall and in localized cortical gray and white matter volumes), possibly due to larger neuronal damage or myelin loss in different brain regions, leading to their poorer cognitive abilities (228). Long-term smoking can add to and synergize with the detrimental effects of HIV infection on DTI (227). Alcohol consumption (even occasional) may exacerbate systemic inflammation, cognitive function, mental health, and brain structural changes (DTI and sMRI) associated with perinatally acquired HIV (PHIV) (229, 230). Apart from the pallidum, chronic marijuana (MJ) use does not have additional adverse effects on brain microstructure or neurocognitive deficits in HIV + individuals (DTI) (231). However, some studies suggest that prior MJ use is associated with similar or improved neurocognitive abilities in various domains when compared to PLWH who have never used MJ (232). Moreover, recent reviews have found that marijuana may have anti-inflammatory effects, potentially offering beneficial interventions to reduce the incidence of inflammation-related morbidities in PWH (233), and it may even protect against the harmful effects of methamphetamine on neurocognitive outcomes (234). In summary, the impact of various clinical factors on HAND is crucial for understanding its pathophysiology and developing precise biomarkers, and we should consider them as comprehensively as possible in our research endeavors.

Moreover, a recent comprehensive assessment of 402 HIV-infected individuals over 12 years in the CNS HIV Antiretroviral Therapy Effects Research (CHARTER) project revealed that the decline in neurocognitive function is not associated with HIV disease or treatment characteristics but significantly correlates with the presence of comorbidities, especially diabetes, hypertension, chronic pulmonary disease, frailty, neuropathic pain, depression, and a history of lifelong cannabis use disorder. These findings are inconsistent with premature or accelerated neurocognitive aging caused by HIV itself, but they suggest that multiple treatable comorbidities have important indirect effects, which are more common in HIV-infected individuals than the general population and may require greater attention in the HIV treatment spectrum (235).

## Combined immunological and imaging biomarkers

Since neurological examinations of HIV patients typically appear normal in the early stages, early diagnosis is challenging but crucial (136). Establishing a CNS HIV repository has lagged behind systemic HIV repositories, presenting an opportunity for early intervention (236). Preliminary studies have shown that neuroinjury markers such as neurofilament light chain protein, tau protein, and amyloid precursor protein do not increase in the early stages of ANI or shortly after cART initiation (144, 237). Instead, these markers typically manifest in the mid to late stages of the disease (237–239), varying with disease severity and decreasing after treatment initiation (238), correlating with low CD4 + T cell counts (140), and MRI markers of neuronal damage (240). The gap between early involvement and clinical HAND symptoms suggests a lengthy treatment window during which targeted interventions can preserve cognitive function. Combining plasma and cerebrospinal fluid immune biomarkers with neuroimaging biomarkers facilitates a better understanding of the mechanisms and potential applications of brain immune regulation. Simultaneous extraction and cross-validation of neuroimaging and immunological biomarkers will contribute to a comprehensive and objective system of predictive biomarkers for HAND. Additionally, future research can utilize these correlated markers to assess and predict the effectiveness of antiretroviral therapy in HAND patients (206).

Morgell et al. combined immunological features with MRI analysis and confirmed a significant association between the two. They found that 1H-MRS neuro-metabolite changes were most frequently predicted by immune factors sensitive to temporal variations, while DWI metrics were more commonly associated with long-term disease status (241). This suggests that white matter microstructural alterations are a long-term process. Finkelstein et al. assessed the correlations between MRI metrics, cognitive performance, and blood markers such as NFL and Tau protein. They established a fusion model by collecting T1-weighted images, diffusion tensor images, neuropsychological assessments, and plasma levels of markers related to neuroinflammation and neurodegeneration, providing a sensitive biomarker to monitor axonal integrity in HIV-infected individuals (242) (Figure 1C).

Not only is the loss of subcortical and white matter volume associated with the lowest CD4 counts, but brain network analysis has also revealed a positive correlation between CD4 counts and gray matter volume in the anterior cingulate cortex and sensorimotor areas, which is further related to lower scores on mental status examinations (163). A higher number of years with CD4 + cell counts <500/μL is also correlated with lower FA and higher MD in the projection, association, and corpus callosum fiber systems (181). The peripheral viral reservoir is associated with HIV-related brain atrophy, with CD14 monocyte cell HIV DNA carriage directly related to brain volume loss (243). Despite the inhibitory effects of ART, elevated HIV-1 transcripts in CSF cells are associated with brain injury (CSF HIV-1 cell-associated (CA)-RNA levels correlated with brain injury in the frontal white matter, posterior cingulate cortex, and the splenium of the corpus callosum). To a lesser extent, this is attributed to reduced neuronal/axonal integrity (reflected by lower NAA/H2O among other brain regions, particularly in the frontal white matter) (63).

Despite viral suppression, inflammatory processes continue to impact clinically relevant brain health indicators (183). Besides the classic implicated regions in the basal ganglia, volume of structures in the limbic system was found to be consistently associated with current plasma biomarkers in a large international HIV-positive population (244). In virally suppressed individuals, elevated inflammatory plasma biomarkers are associated with DTI white matter abnormalities (183). Vascular endothelial growth factor (VEGF) and macrophage inflammatory proteins (MIP) 1β and 1α are consistently correlated with low FA and high MD in white matter tracts. Elevated serum levels of VEGF, MIP-1α, and MIP-1β are associated with alterations in brain white matter microstructure (245). These blood biomarkers may aid in predicting HIV-related white matter damage. Nguchu et al. utilized T1-weighted anatomical images, diffusion tensor imaging along the perivascular space (DTI-ALPS), and blood CD4 + T cell counts and CD4+/CD8 + ratios to study the lymphatic system status in HIV-infected individuals. They demonstrated that HIV can cause the presence of HIV-induced changes in the glymphatic flow, which may reflect compensatory mechanisms of the microenvironment homeostasis in response to HIV damage. This elucidates the clinical significance of assessing lymphatic system function based on ALPS index and suggests that improving the lymphatic system could be an alternative treatment strategy for HAND (246).

Plasma levels of sCD14 and sTNF-RII are elevated in patients with cognitive impairment and brain atrophy, and both factors are correlated with spectroscopic choline:creatine ratios (247). IP-10 and MCP-1 are chemokines most closely associated with individual brain metabolite levels (248). Increased plasma or CSF sCD14 and MCP-1 are linked to reduced NAA/Cr levels in the frontal cortex (MFC), frontal white matter, and basal ganglia (BG) as measured by spectroscopic MRS; higher CSF FKN is associated with increased NAA/Cr in the BG; plasma and CSF IP-10 correlate with Cho/Cr in the MFC (249). These findings support the idea that peripheral immune responses are related to cognitive dysfunction during HIV infection and metabolite levels. During acute HIV infection, CSF NFL levels are negatively correlated with neuroimaging markers of neuronal health (N-acetylaspartate/creatine) levels, and these correlations persist after treatment (144). In people living with HIV-1 infection (PLHIV) after suppressive ART, CSF cell-associated (CA) HIV-RNA levels correlate with damage in the frontal white matter (FWM), posterior cingulate cortex (PCC), and caudate nucleus as measured by 1H MRS (63). This is driven by reduced neuronal/axonal integrity reflected in lower NAA/H2O levels in the FWM and other brain areas. In a study of chRMs infected with SIV, even with combination ART, the basal ganglia had a higher frequency of SIV carriage than other brain regions (80), and reduced activated CD14 + CD16 + monocytes were associated with reversal of neuronal damage as measured by NAA/Cr levels (250). These changes suggest that brain damage caused by metabolite and region-dependent means may be irreparable in chronically HIV-infected patients under stable ART treatment. Pre-treatment neuroinflammatory changes in the basal ganglia and thalamus of PWH patients (significantly elevated FDG uptake values) decreased after treatment, revealing long-term irreversible neuronal damage. In the thalamus, changes in FDG uptake correlated with changes in CD4 + cell counts and viral load and were associated with IL-6R and sCD14 (212).

Assessment of regional brain volume changes in individuals who initiated cART treatment within weeks of HIV exposure and evaluated at 24 months reveals that volume loss of the caudate and putamen are associated with the early expansion of monocytes expressing P-selectin glycoprotein ligand-1 (PSGL-1), particularly intermediate/inflammatory subpopulations. Initiating suppressive cART during AHI may not prevent brain atrophy, and potential mechanisms for progressive HIV-related atrophy may include early activation of circulating monocyte populations and enhanced adhesion and migratory capabilities (251). Despite treatment, immune activation is believed to result in perinatal HIV-associated brain injury in children. Recent research has, for the first time, concurrently assessed the relationship between plasma and cerebrospinal fluid soluble biomarkers, neuroimaging abnormalities, and cognitive function in this specific population. It was found that plasma C-reactive protein, interferon-*γ*, interferon-γ-induced protein-10, and monocyte chemoattractant protein-1 levels were elevated in HIV-infected children compared to the control group. Among HIV-infected participants, plasma soluble CD14 was positively correlated with white matter (WM) microstructural damage, and plasma D-dimer was negatively correlated with WM perfusion. In the CSF, IL-6 was negatively correlated with WM volume, and neurofilament heavy chain (NFH) was negatively correlated with IQ and working memory. These biomarkers of persistent inflammation, immune activation, coagulation, and neuronal injury can be used to further evaluate the pathophysiology and clinical course of brain and cognitive deficits in perinatally acquired HIV patients (252).

Maraviroc is an FDA-approved antiretroviral drug that operates by blocking the C-C chemokine receptor 5 (CCR5), thus impeding HIV entry into cells. It is conjectured that the blockade of the CCR5 chemokine receptor also exhibits anti-inflammatory properties. Within a cohort of HIV-infected individuals undergoing intensified antiretroviral therapy with maraviroc, an increase in NAA concentration within the basal ganglia was observed, which correlated with maraviroc plasma concentrations. This correlation is likely associated with changes in the inflammatory milieu within the central nervous system (253). A phase 1 pharmacokinetic and 1H-MRS study conducted on 12 asymptomatic adults with HIV infection revealed a significant correlation between cerebrospinal fluid chemokines IP-10, MCP-4, and MIP-1β, and cerebral metabolite ratios (CMRs) in the right basal ganglia. This suggests a direct cerebral effect of maraviroc. When compared to other brain regions, the basal ganglia exhibit higher blood flow and a more permeable blood–brain barrier, implying earlier exposure to plasma drugs in this region (206).

Suzuki et al. employed highly sensitive Double R assays to detect and quantify HIV-1 DNA copy numbers and cell-associated (CA) HIV-1 RNA copy numbers in the cerebrospinal fluid and peripheral blood mononuclear cells (pbmc) of PLHIV (people living with HIV) under suppressive antiretroviral therapy. They then correlated these findings with brain damage assessed by 1H MRS. Their findings revealed that despite effective viral suppression through antiretroviral therapy, HIV RNA transcription levels remained high within cerebrospinal fluid cells, which were associated with ongoing brain damage. These results challenge the prevailing notion that the neuropathogenesis of HIV is a consequence of residual damage caused by prior or concurrent comorbidities. Currently available antiretroviral drugs do not inhibit the transcription stage of the HIV life cycle. Furthermore, the transcriptional load of HIV in monocytes is much lower than that in CD4 T cells (HIV DNA or HIV CA-RNA is nearly undetectable in monocytes in peripheral blood, suggesting their limited involvement in transporting infected cells into the central nervous system), thus weakening the conventional perspective that infected monocytes in circulation are the cause of HIV neuropathogenesis. These results underscore the need for novel drugs targeting transcription (63).

**Figure 2:**
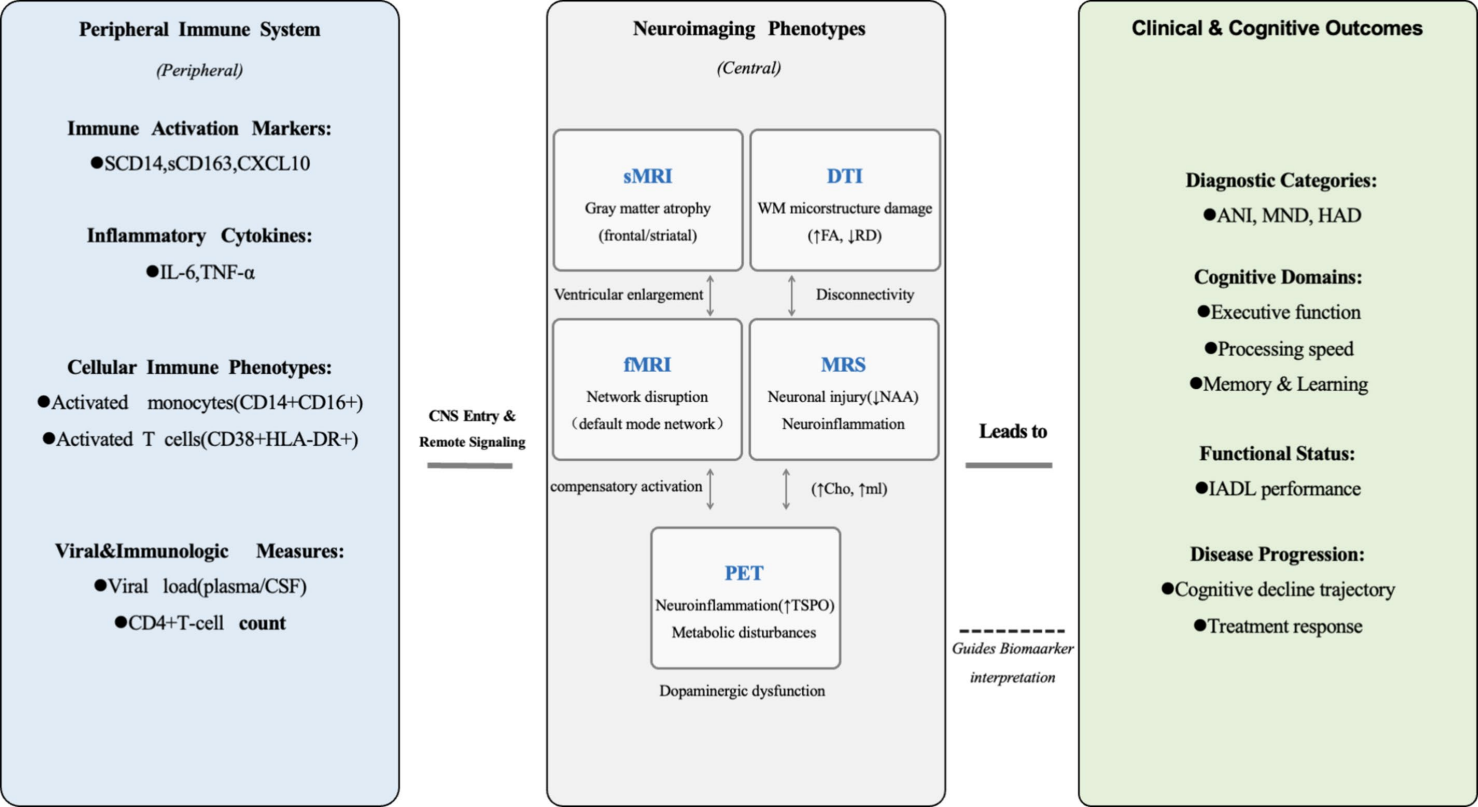
Integrative biomarker framework for NeuroHIV: Linking peripheral immunity, neuroimaging phenotypes, and clinical outcomes. This conceptual figure illustrates the proposed integrative framework linking peripheral immune dysregulation, multi-modal neuroimaging phenotypes, and clinical outcomes in people living with HIV. The arrows indicate hypothesized directions of influence. The framework highlights the need for future studies to move beyond associative analyses toward causal integrative models for improved diagnosis and prognosis.

It is evident that ongoing research in immunological mechanisms continues to provide fresh perspectives on the treatment of HIV-associated neurocognitive impairments. Simultaneously, advances in neuroimaging techniques for HIV-associated neurocognitive impairments are making significant strides. Research into immune mechanisms continues to provide new insights for the treatment of HIV-related cognitive impairment. The integration of immunological and neuroimaging biomarkers—through approaches such as scoring systems, staging systems, or algorithms—will be a focal point for future studies (36). This research offers investigators a straightforward integrated system for reference (Table 1). To this end, we have designed a conceptual figure that visualizes this integrative framework, illustrating the connections between peripheral immune dysregulation, multimodal neuroimaging phenotypes, and clinical cognitive outcomes (Figure 2).

**Table 1:** Immunological and imaging biomarkers with recommended strengths.

Approach		Indicator	Key findings	Recommended strength
Immunological biomarkers in blood and CSF	Immune cells	lymphocytes	In HAND patients, blood CD4 + and CD8 + T cells exhibit active viral transcription (62, 64). Compared to blood, the frequency of dendritic cells in the cerebrospinal fluid of people with HIV (PWH) is increased, while the frequencies of B cells and natural killer (NK) cells are decreased (68).	Moderate
		Myeloid lineage cells	Monocyte and macrophage populations are increased (69, 71). Compared to blood, cerebrospinal fluid (CSF) contains a higher proportion of CD8 + T cells and microglia-like cells (72, 73).	Moderate
	Cytokines		Specific monocyte activation markers in blood and cerebrospinal fluid, such as elevated levels of new methotrexate, sCD163, and sCD14, along with neuroinflammation markers, including increased levels of IFN-*γ*, IL-1α, IL-7, IL-8, and sTNFR-II, as well as decreased levels of IL-6, demonstrate a consistent association with HIV-related neurocognitive impairment (49).	Moderate
	Complement		In HIV patients, levels of C3 protein, CD55, CD59, C9, C5L2, C5aR, and C3aR are elevated (66, 122, 124).	Moderate
	Immunoglobulins and antigens		HIV proteins Tat, gp120, and Nef are closely associated with HAND, and patients with HAND exhibit inflammatory changes in their brain antibody profiles (133–135).	Moderate
	Others		Elevated concentrations of neurofilament light chain protein (NFL), new methotrexate, extracellular vesicles, β-amyloid, tau protein, and S100B protein are closely associated with poorer neuropsychological performance (30, 78, 80, 138, 143, 146).	Moderate
CT		Macroscopic brain structure	Diffuse brain atrophy inconsistent with age in individuals with HIV (165).	Weak
MRI	3D- TIWI	Microscopic brain gray matter structure (volume, cortical thickness, etc.)	Reduced volume of the frontal lobe and caudate/putamen is associated with the disease itself and neurocognitive impairments (171, 172).	Strong
	DTI	Fractional anisotropy (FA), mean diffusivity (MD), axial diffusivity (AD), and radial diffusivity (RD)	Significantly increased MD and decreased FA in the right corpus callosum, corona radiata, internal capsule, and posterior thalamus are significantly associated with poorer cognitive performance (178, 180–182).	Strong
	rsfMRI	BOLD signal	Alterations in global brain topology are observed, with significant reductions in the significance and connectivity of the executive network (190–192).	Strong
	Task-based MRI	/	Different task difficulties activate distinct brain regions, and abnormal activation may reflect a compensatory mechanism (194, 196–199).	Strong
	MRS	NAA Peak, Glu Peak, Cho Peak, MI Peak, etc.	Decreased NAA and elevated Cho and MI levels are primarily observed in the frontal white matter and basal ganglia. Prolonged duration of HIV infection is associated with reduced NAA levels in the caudate nucleus (201–208).	Strong
Radioisotopes	[18F]-FDG PET	SUVR	Cortical hypometabolic areas alongside subcortical regions (including the basal ganglia and thalamus) are early features of HIV-associated dementia (211, 213).	Moderate
	18 kDa (TSPO)	SUVR	There is a significant increase in expression in subcortical gray matter, particularly in the basal ganglia, including the globus pallidus, caudate nucleus, and putamen (214).	
	[11C]-(R)- PK11195	SUVR	In HIV patients, there is a significant increase in [iC]-R-PK11195 binding in the thalamus, putamen, cerebellum, frontal cortex, and occipital cortex (217).	Moderate

CSF, cerebrospinal fluid; CT, computed tomography; MRI, magnetic resonance imaging; DTI, diffusion tensor imaging; rsfMRI, resting-state fMRI; MRS, magnetic resonance spectroscopy; NAA, N-acetyl aspartate; Glu, Glutamic acid; Cho, Choline; MI, myoinositol; PET, positron emission tomography; SUVR, standardized uptake value ratios.

## Special considerations in elderly PWH

In elderly people with HIV (PWH), the interplay between immunosenescence and chronic viral infection forms a distinct backdrop for neurological health. This population often exhibits features of accelerated immunosenescence, where a history of severe immunosuppression—marked by a low nadir CD4 + count—and a persistent state of chronic immune activation remain closely associated with neuroimaging evidence of structural brain injury, even under effective viral suppression (244, 254). Neuroimaging research provides critical evidence for this: structural MRI reveals that elderly PWH frequently demonstrate patterns of brain atrophy extending beyond normal aging, particularly in subcortical and limbic regions, which correlate with cognitive decline (255). Diffusion MRI further uncovers widespread microstructural damage to white matter, while functional MRI identifies altered patterns of brain network activation and reduced functional connectivity efficiency (256). Moreover, advanced techniques like dynamic contrast-enhanced MRI (DCE-MRI) have confirmed compromised blood–brain barrier integrity in some patients, representing a potential mechanism for sustained neuroinflammation (257). To date, positron emission tomography (PET) studies have not consistently identified significant Alzheimer’s disease-like amyloid deposition in virologically suppressed elderly PWH, aiding clinical differential diagnosis (258). In summary, multimodal neuroimaging offers a vital window into the unique trajectory of brain aging in elderly PWH, playing an indispensable role in monitoring neurological injury, differentiating causes of cognitive impairment, and evaluating therapeutic interventions.

## Treatment

The widespread use of combination antiretroviral therapy (cART) makes it more critical than ever to consider HAND treatment, particularly for aging HIV patients who, despite being on cART for many years or even decades, continue to experience persistent systemic and central nervous system inflammation. Developing effective biomarkers or clinical neurocognitive tests that can accurately stratify the risk of HAND is a crucial step toward improving treatment. In addition to cART, targeting inflammation processes that are independent of viral load with immunomodulatory drugs appears to be a promising approach to reducing chronic inflammation and maintaining cognitive function in HAND patients. Given the extended life expectancy of HIV-positive individuals, understanding the pathogenic mechanisms that lead to cognitive impairment and addressing patient burden with new therapeutic strategies is becoming increasingly important.

In addition, there are various potential therapeutic approaches targeting multiple key pathogenic processes in HAND. These include blocking the transport of lymphocytes into the central nervous system, reducing the transport of monocytes, modifying NFk *β* through the reduction of viral replication, and decreasing the activation of microglial cells (35). Research also emphasizes the importance of targeting activated monocytes and macrophages as key players in SIV and HIV infections, suggesting that adjunctive therapies targeting monocytes/macrophages may prevent or reduce CNS pathology associated with HAND (259). JAM-A and ALCAM, which reduce CD14(+)CD16(+) monocytes, are potential therapeutic targets for preventing these cells from entering the central nervous system of HIV-seropositive individuals, thereby contributing to the eradication of neuroinflammation, HAND, and the CNS viral reservoir (29). Additionally, research has shown that, under ART, HIV-infected memory CD4 T cells represent a unique population with a host gene expression profile that promotes HIV latency, cell survival, and proliferation, highlighting their significance in the development of HIV treatment strategies (260). Nef has been shown to play a role in preventing the clearance of HIV-1 infected cells, and Nef inhibitors can have a supportive role in various treatment modalities, including shock and kill strategies, as well as HIV-1 immunotherapies such as therapeutic vaccine administration and CAR-T cell therapy (261). Recent studies have also identified PLK1 as a promising target for chemically “killing” HIV-1 reservoir cells (262). Moreover, adjunctive neuroprotective strategies, which combine existing FDA-approved drugs, cognitive therapy, aerobic exercise, and improved cART, offer a rational approach to optimizing the prevention and treatment of HAND (263). Additionally, molecular neuroimaging may continue to serve as a non-invasive tool for evaluating neuroinflammation (neuroimmune changes) and monitoring changes in the central nervous system’s response to therapy (36).

However, certain antiretroviral therapies (ART) have been shown to have neurotoxic effects (264). The behavior of the immune system within the central nervous system is unpredictable; reports indicate that up to 13% of HIV patients may develop HIV-associated immune reconstitution inflammatory syndrome after initiating cART, suggesting that immune recovery, particularly the restoration of CD4 T cells, can lead to complications in the central nervous system (265). To optimize the beneficial effects of treatment, it is essential to consider the balance between the health risks and benefits of ART. While the most toxic antiretroviral drugs, such as efavirenz, are gradually being phased out, further research is needed to thoroughly differentiate the toxicity of antiretroviral therapies and their impact on comorbidities in HIV-positive patients (266).

## Conclusion and future perspective

In 2015, NEJM advocated for the initiation of antiretroviral therapy (ART) as soon as possible, regardless of CD4 T-cell counts, highlighting the benefits outweighing the risks for both infected individuals and uninfected sexual partners. This approach was recommended to be implemented in a patient-acceptable manner, effectively preventing HIV transmission (267). Numerous studies have underscored the importance of early ART in mitigating the long-term impact of HIV infection on neurocognition (268). Any of the first-line antiretroviral regimens recommended by the World Health Organization has been shown to enhance neurocognitive function and reduce neurocognitive impairments (269).

In summary, the early development of effective immune and imaging combined biomarkers will aid in accurately stratifying the risk of HAND, representing a crucial step toward improving treatment. Considering the increasing life expectancy of HIV patients, the importance of understanding the pathogenic mechanisms leading to cognitive impairment and employing novel treatment approaches to alleviate the burden on patients cannot be overemphasized. In addition to cART, the use of immune-modulating drugs targeted at inflammation processes independent of viral load, along with the precise control and monitoring of immune drug therapy using innovative immune and imaging combined biomarkers, appears to be an intriguing and effective strategy for reducing chronic inflammation and maintaining cognitive function in HAND patients.

## Data Availability

The raw data supporting the conclusions of this article will be made available by the authors. Requests to access the datasets should be directed to Xing-Yuan Jiang, xyjiang_0112@163.com.
